# Fitness consequences of biochemical adaptation in *Drosophila melanogaster* populations under simultaneous selection for faster pre-adult development and extended lifespan

**DOI:** 10.1038/s41598-021-95951-2

**Published:** 2021-08-12

**Authors:** Khushboo Sharma, Mallikarjun N. Shakarad

**Affiliations:** Evolutionary Biology Laboratory, Department of Zoology, University of Delhi, Delhi, India

**Keywords:** Experimental evolution, Evolution, Zoology, Entomology

## Abstract

In holometabolous insects like *Drosophila melanogaster*, critical size is an important time point during larval life, for irreversible commitment to metamorphosis. Here, we studied the impact of restricted growth duration in terms of selection for faster pre-adult development in *Drosophila melanogaster* populations which resulted in the evolution of reduced critical size on adult life history traits. Selection for faster pre-adult development resulted in biochemical adaptation in larval physiology with no compromise in major biomolecules at critical size time point. The flies from the selected populations seem to not only commit to metamorphosis on the attainment of critical size but also seem to channelize resources to reproduction as indicated by similar life-time fecundity of CS and NS flies from selected populations, while the Control CS flies significantly lower life-time fecundity compared to Control NS flies. The flies from selected populations seem to achieve longevity comparable to control flies despite being significantly smaller in size-thus resource constrained due to faster pre-adult development.

## Introduction

Early life nourishment is known to affect the adult physiology and various life-history traits in *Drosophila melanogaster*^1–3^ and other holometabolous insects^4^. In *D. melanogaster*, early life is divided into pre-adult duration consisting of mobile and voraciously feeding larval phase and non-mobile pupal phase. Further, the larval phase is marked into two stages—(i) pre-critical stage and (ii) post-critical (terminal growth period) stage, separated by critical size time point^5,6^. Larval critical size commit the larva to an irreversible process of metamorphosis, and starvation post attainment of critical size does not affect time course to undergo metamorphosis^5,7^. The role of critical size as “physiological switch” is well established and is marked by ecdysone pulse^7^. Further, critical size is reported to also act as “energy allocation switch” in various species of *Drosophila*^8,9^. In another dipteran species, *Aedes egypti*, it has been previously reported that threshold amount of energy reserves are pre-requisite to the process of metamorphosis in addition to ecdysone level during last larval instar, thus implying significance of energy budgeting in the dipteran species^10^.

‘Developmental threshold model’ states that there is always a minimum size or condition that must be surpassed before the life-history transition occurs in a wide variety of species undergoing metamorphosis^11,12^. While larger threshold causes a negative relationship between age of transition and growth conditions, the smaller threshold would result in a comparatively positive relationship for higher growth rate. In fast-developing individuals, once this threshold is passed then the excess of energy or resource is translated to overhead threshold and invested in fecundity^11^.

In *Drosophila melanogaster*, this minimum developmental threshold is represented by critical size/time point^5,6,13^. Being an inhabitant of rotting fruits and vegetables, it is under direct selection for faster pre-adult development due to over-crowding and food limitation. Selection for faster pre-adult development is known to exhibit reduced development time and subsequently results in the smaller adult body size^13–18^. In *D. melanogaster,* environmental conditions during larval life are suggested to affect its adult size^19^ and adult physiology^19,20^ which in turn affect various life-history traits^1,3,21–24^. Further, if the larval nutritional environment or developmental diet is rich then they tend to emerge with larger adult body size and attain reproductive maturity at an early age that has a positive implication on fitness^11,25,26^. In general though not universal^18^ “bigger is better” idea prevails with larvae spending more time in weight (equal to resource/energy) gain under good nourishment conditions, eventually emerging as larger adults with higher fitness. Also ‘Silver-spoon hypothesis^25,27^’ posits that individuals born in good conditions have fitness or performance advantages in later life with many examples^25,27^ and those born in poor conditions are at a permanent disadvantage^27^. Varying the quality or quantity of diet during development in *D. melanogaster* affects adult body size and its associated life history, hence it is known to serve as a tractable model to study dietary manipulation during pre-adult and adult stages^24,28,29^.

The inverse relationship between time to maturity (metamorphosis) and size is expected in species that live in ephemeral habitat^12^. A physiological change from resource-dependent (pre-critical) to resource independent (post-critical duration) rate of development occurs at critical size or threshold time point^7^. Further, the quality of nutrition during developmental stages affect adult life history traits^30–33^. Furthermore, quality of food during developmental stage is also known to result in short term adaptive response where in females that were deprived of yeast diet as larvae had reduced fecundity but mortality rate was unaltered^24^ suggesting that longevity-fecundity trade-off may not be universal.

In the present study, we demonstrate the significance of critical size/threshold size that commit holometabolous insect like *Drosophila melanogaster* to irreversible process of metamorphosis^5,7^, on larval physiology and adult life history traits in populations under simultaneous selection for faster pre-adult development and extended lifespan*.* Of the six, *Drosophila melanogaster* populations used in this study, three-selected populations were internally driven to stop feeding in about 17-h post attainment of critical size thus curtail food intake, while the ancestral three-control populations fed for 42-h post attainment of critical size though they had similar food intake capacity during larval life^13^. We observed the biochemical adaptations in larval physiology of selected populations. There was comparable glycogen level during third larval instar while level of lipid and protein content were reduced during post-critical duration though similar at critical size time point due to selection for faster pre-adult development. The realized life-time fecundity of the populations selected for faster pre-adult development was significantly different when allowed to feed till natural pupation compared to the control populations under similar conditions. Interestingly, within the selected populations, the fecundity of the adult flies that had fed only till attainment of critical size thus under curtailed food in-take was comparable to those that fed up to their natural pupation time suggesting that the flies from the selected populations were committing the available energy reserves to reproduction while committing themselves to irreversible process of metamorphosis.

## Results

We subjected each trait data to Shapiro–Wilk normality test (data not shown). The protein content, lipid content and total energy data were non-normal and hence were subjected to log-transformations. For all data that passed the normality test, Bartlett's test was carried out to assess homogeneity of variance. The results indicate *homoscedasity* (see Supplementary Table S1). Following this, each trait data was subjected to factorial analysis of variance (ANOVA) with selection, larval feeding duration and gender as fixed factors, and replications as random factors as in Prasad et al.^17^, unless mentioned otherwise. Since, in all cases, the population means were used as the units of analysis, only fixed-factor effects and interactions could be tested for significance^17^.

### Biochemical assays during larval life

**Figure 1:**
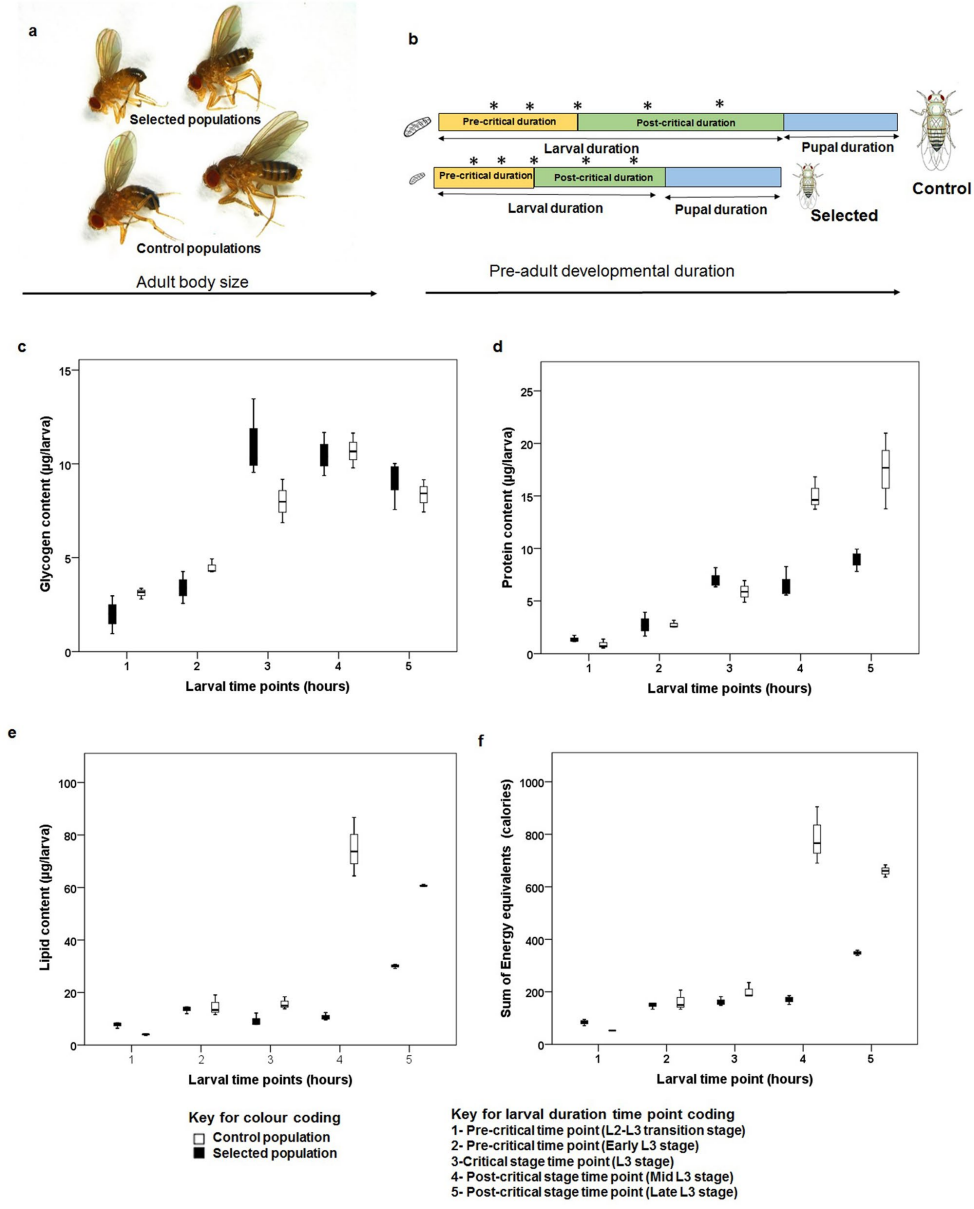
(**a**) Representative images of male and female flies from selected and control populations. (**b**) Schematics of protocol followed for larval biomolecule assay time points in selected and control populations. (**c**) Glycogen content (µg) per larva, (**d**) protein content (µg) per larva, (**e**) lipid content (µg) per larva, and (**f**) sum of energy equivalent (calories). Box plot is depicting the median value along with lower and upper quartile and the whiskers indicate the minimum and maximum values. *In (**b**) indicate the larval sampling time points.

There was no significant effect of selection (F_1,20_ = 0.481, p = 0.496, Fig. 1c) on glycogen levels. However, there was a highly significant effect of larval growth time (F_4,20_ = 62.678, p = 0.000) on glycogen content (Fig. 1c). Further, selection × larval growth time interaction was also significant (F_4,20_ = 3.724, p = 0.02). Protein and lipid content, and total energy level traits failed the normality test and hence were log-transformed, validated for normality and then subjected to factorial ANOVA. Interestingly, selection (F_1,20_ = 3.551, p = 0.074) showed no significant effect on protein content. However, larval growth time (F_4,20_ = 95.957, p = 0.000) and selection × larval growth time interaction (F_4,20_ = 7.466, p = 0.001) had significant effect on protein content (Fig. 1d). Similarly, selection (F_1,20_ = 85.446, p = 0.000), larval growth time (F_4,20_ = 161.565, p = 0.000) and selection × larval growth time interaction (F_4,20_ = 58.42, p = 0.000) had significant effect on lipid content(Fig. 1e). The energy equivalents of the biomolecules were summed and log-transformed before the effects of selection, larval growth time and their interaction were ascertained. There was a significant effect of selection (F_1,20_ = 85.143, p < 0.000; Fig. 1f), larval growth time (F_4,20_ = 256.753, p < 0.000; Fig. 1f) and selection × larval growth time interaction (F_4,20_ = 58.338, p < 0.000).

### Realized life-time fecundity and dry weight of flies

**Figure 2:**
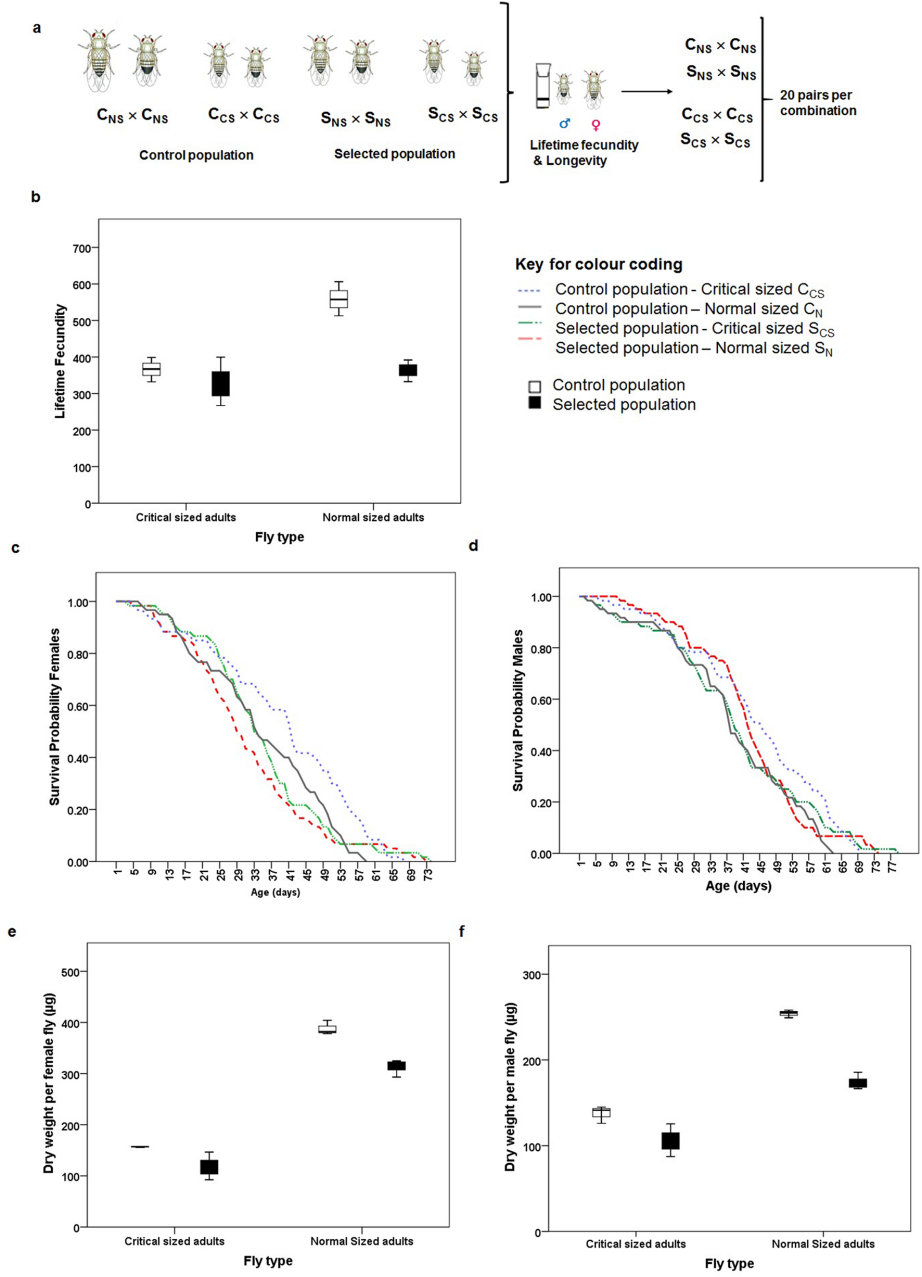
(**a**) Specifications of pairs of flies for longevity and realized life-time fecundity assay set up. (**b**) realized life-time fecundity of selected and control population under critical size and normal size conditions, (**c**) survival probability of females, (**d**) survival probability of males, (e) dry weight (µg) per female fly, and (**f**) dry weight (µg) per male fly. Box plot is depicting the median value along with lower and upper quartile and the whiskers indicate the minimum and maximum values.

There was equal variance among all the dataset in present assay (see Supplementary Table S1). There was a significant effect of selection (F_1,8_ = 18.772, p = 0.003, Fig. 2b) on realized life-time fecundity. We observed significant effect of fly type on realized fecundity with flies that emerged from larvae fed up to critical time point showing significantly lower fecundity (F_1,8_ = 17.972, p = 0.003, Fig. 2b). Further, there was selection × fly type interaction effect (F_1,8_ = 8.731, p = 0.018). Fitting a regression model to fecundity as a function of female dry weight using the data from control critical size and normal flies yielded, fecundity = 232.438 + (0.844 × female dry weight). The average weight of the selected critical size female flies was 117.876 μg and that of normal size female flies was 312.996 μg. Using the regression from control flies, the predicted fecundity of the selected critical size and normal size flies would be 331.925 and 496.607 respectively. The observed fecundity of the selected normal flies was significantly lower than expected from regression model (χ^2^ = 35.748, p < 0.001). Three way ANOVA with selection, fly type (feeding only till critical time point or feeding till natural pupation) and gender as independent variables and dry weight as dependent variable indicated significant effect of selection (F_1,16_ = 83.635, p < 0.000), gender (F_1,16_ = 151.831, p < 0.000) and fly type (F_1,16_ = 613.097, p < 0.000). However selection × gender (F_1,16_ = 0.006, p = 0.939) and selection × gender × fly type (F_1,16_ = 0.246, p = 0.627) interactions were not significant.

### Longevity and survival probability

There is no significant effect of selection and diet curtailing post attainment of critical size on average longevity of both male and female flies. Overall, CS females had significantly higher median longevity than NS flies. However, there was no significant effect of selection on median longevity. The maximum lifespan of critical-sized males was significantly altered (see Supplementary Table S2).

Average, median and maximum longevity are descriptive statistics that can at best provide overall comparison. Since our sample sizes are small compared to those of Rose et al.^48^ hence we compared the survival probabilities using Kaplan–Meier Analysis—a non parametric test to assess age specific survival rates^34^. There was a significant effect of selection and fly type on survival probabilities of both male and female flies (see Table 1, Fig. 2c,d).

**Table 1:** Comparison of age dependent survival of males and females under selection for faster pre-adult development and extended longevity, and larval diet curtailing at critical size.

Population and fly type	Female			Male		
	S_NS_ and C_NS_	S_CS_ and S_NS_	C_CS_ and C_NS_	S_NS_ and C_NS_	S_CS_ and S_NS_	C_CS_ and C_NS_
χ^2^	47.78	47.67	31.38	21.87	20.41	20.10
P	< 0.001	< 0.001	< 0.001	< 0.001	< 0.001	< 0.001

## Discussion

Recently, it has been reported that glycogen metabolism is required during third larval instar for normal body size growth and even the developmental delay would not rescue the arrest of body size due to reduced glycogen levels in the larval stage^35^. Further, it has been evidenced that defects in glycogen metabolism are known to affect larval physiology and hence the adult fitness^35^. While the fat body, muscles and Central Nervous System (CNS) in larva act as the site for glycogen storage^36^, glycogen synthesis occurs during late larval life in fat body^36,37^. Furthermore, fat bodies act as a peripheral system for ecdysone metabolism^38^. Despite smaller larval size than controls^39^, the selected populations had comparable levels of glycogen (Fig. 1c, Supplementary Table S4) throughout the third instar suggesting that glycogen is the primary source of energy that is possibly driving the physiological processes leading to early expression and release of ecdysone in selected populations^39^ that might in turn facilitate faster development.

When we compared the trend of lipid content during larval life, it is comparable at first (48 h post-hatching of synchronized eggs), 2nd (early L3) and 3rd larval time point (Critical size time point-developmental threshold/metamorphosis commitment time point) suggesting the utilization of lipids for metabolic processes during these periods (see Supplementary Table S4).The lower level of lipid post-critical size attainment at 4th and 5th larval time point is perhaps due to drastic decrease in the post-critical growth duration of the selected populations^13^. It is known that lipids are majorly stored in fat body and utilized during the pupal stage and early adult life^23^. The energy equivalent (in terms of calories) of lipids are highest (see Supplementary Fig. 1c) among the three macromolecules. Selected populations undergo early metamorphosis without compromising on pupal duration^13^ despite having lower energy levels towards the end of the larval phase, suggesting that the energy levels up until critical time point might be channelized towards metamorphosis.

There was marginal effect of selection (p = 0.074) on the protein content. The comparable protein levels at 1st, 2nd and 3rd time point representing the time points of higher wet weight gain (see Supplementary Table S4) could be responsible for the marginal effect. The low protein level at 4th and 5th larval time point in selected populations might be due to the utilization of proteins in the growth and development of imaginal discs^39^. It is likely that as protein content during late larval life in selected populations is catabolized for preparing the organism for metamorphosis while the control flies continue to accumulate proteins during nearly 40 h long post-critical duration^13^. For example, proteins like Larval serum protein (LSP) increases drastically in the third larval stage, thus making it the most abundant protein during the post-critical duration that contributes to the major wet weight of larva^40^ in our control populations.

The major macromolecules and energy results indicate that our selected populations have evolved mechanisms to maintain the necessary levels of molecules (and energy) till attainment of critical size-a time point at which the organisms commit to an irreversible process of metamorphosis. This is in agreement with Hironaka et al.^8^ who proposed that the investment value in larval tissue is more during the pre-critical period than terminal growth period (a.k.a. post-critical duration)^8^. Further, supporting the view that critical size in *Drosophila melanogaster* is acting as an optimal switch for energy allocation during larval life^8,9^. Furthermore, following the model for developmental threshold- with the age of maturity (metamorphosis-in the present context) and size at transition, it is likely that selection for faster pre-adult development evolved smaller developmental threshold (critical size-in this case) through higher growth rate during second larval instar^13^ thus accumulating sufficient energy reserves to sustain the development without compromising on their metamorphosis duration^11,13^. Although the selected populations are under the physiological trigger to complete development due to higher ecdysone levels throughout L3^39^ they can accumulate adequate proteins and lipids to maintain their phenotypic integrity albeit emerge as small adults^13,39^.

A developmental dietary history is known to influence adult physiology^1^. In general, the trade-off between longevity and life-time fecundity are well documented in *Drosophila melanogaster*^28,41–45^ and other holometabolous insects like *Speyeria mormonia*^46^. Consistent with dietary restriction studies, the control CS flies had lower fecundity (Fig. 2b). However, exception to larval dietary manipulation has also been reported. For example in holometabolous Lepidopteran butterfly, *Speyeria mormonia*, there was no independent effect of semi-starvation on realized egg laying^45^ suggesting an indirect effect of larval dietary restriction on fecundity. In our study, selection for faster pre-adult development that resulted in small-sized adults (Fig. 2e,f) had significantly reduced life-time fecundity (Fig. 2b, Supplementary Table S3) perhaps due to small sized ovaries^18^ that in turn could have affected the total realized life-time fecundity. Interestingly the life-time fecundity of the critical size flies from the selected and control populations were comparable suggesting that flies might be committing certain amount of resources to reproduction at critical time point. Further, the significantly higher fecundity of normal control flies certainly seems to be due their increased post-critical feeding period. Our results are in agreement with Min et al.^2^ where they reported the contribution of larval resources in early life fecundity in addition to adult diet^2^. However, a recent study reported reduced reproductive fitness as a consequence of small adult size due to dietary manipulation during larval growth period^47^. Further, in agreement with Klepsatel et al.^47^, our selected populations had reduced life-time fecundity owing to their small adult size perhaps due to drastic reduction in post-critical feeding duration and thus represent the cost of rapid development.

The selected populations which is under direct selection for faster pre-adult development and reproduction at unspecified advanced age for more than 134 generations does not show increase in longevity despite ~ 33 day difference in the last age at which reproduction occurs in the selected populations compared to their ancestral controls. This is in contrast to previous experimental evolution studies that have exerted this level of selection for rapid development on age-at-reproduction and reported dramatic decrease of ~ 16 days in mortality-rate plateaus of ACO populations compared to CO populations^48^. They reported a 14–18 day difference in average longevity with only a 20 day difference in age-at-reproduction. While, in present study, the female control population shows a mean longevity that is 3–5 days higher than the selected populations (Fig. 2, Supplementary Fig. S2 and Supplementary Table S2) suggesting yet another cost the selected populations are paying for rapid development.

The reduction in life-time fecundity was accompanied by significant differences in survival probability of females that had emerged from larvae that fed up to critical size than those that had fed till their natural pupation time. This is in sharp contrast to the study on *Speyeria mormonia* where larval food restriction resulted in smaller adults and larval feeding duration affected lifespan but not realized fecundity^46^. A recent study reported the non-universal nature of the trade-off between longevity and fecundity^29^. However, conscious curtailing of diet duration post attainment of critical size in larval life resulted in significant difference in adult survival probability. The direct selection for faster pre-adult development for more than 134 generations has led to the evolution of smaller adult size through reduced critical size^13^ while indirect selection for late reproduction might be responsible for non-significant difference in life span exhibiting limited trade-off^44^. Further, early attainment of high ecdysteroids titer and large Prothoracic gland at comparable time points during larval development in selected populations might be the reason for the evolution of small adult body size and reduced developmental duration^39^.

## Conclusion

Overall, our study provides insight into the role of critical size on adult life-history in *Drosophila melanogaster* populations. The populations that are under simultaneous selection for faster pre-adult development and thus under curtailed food intake, perhaps commit their energy to reproduction and adult longevity immediately on attainment of critical size as indicated by comparable life-time fecundity and increased survival probability of flies that emerged from larvae fed till critical size as opposed to those that fed till natural pupation. However, the flies from the control population that emerged from larvae fed up to critical size had reduced life-time fecundity compared to those that emerged from larvae fed till natural pupation time. Taken together, the selected populations had evolved their physiology to commit available resources to adult fitness at the time of committing to irreversible process of metamorphosis.

## Methods

### Fly husbandry

Two kinds of laboratory *Drosophila melanogaster* populations were used in this study. The Control (C) populations were on 21 days egg to egg discrete generation cycle, while the Selected (S) populations were derived from the controls by direct selection for faster pre-adult development and indirect selection for extended longevity (Fig. 1a,b). Detailed protocols adopted in rearing and maintenance of C and S populations are explained previously^13^. Briefly, each of the three C populations were cultured in 40 vials with 6 mL standard banana-jaggery media (SM) at a density of 40–50 eggs per vial and incubated at Standard Laboratory Conditions (SLC)^13^ for full 12 days^13^. At the end of 12 days, all emerging flies from the 40 vials were transferred to pre-labeled plexi-glass population cages and provided with fresh food every alternate day till day 18. On day 18, fresh food plate was supplemented with live yeast-acetic acid paste. Eggs for initiating the next generation were collected on day 21 from the previous egg collection day. Each of the three S populations was derived from the three C populations by collecting 160 vials of 60–80 eggs per 6 mL banana-jaggery food vial. The vials were incubated at SLC. The early emerging 15–20 flies (as ascertained by empty pupal cases) from each vial were transferred to pre-labeled population cages. Two sister cages were maintained per S population to avoid adult crowding. The S population cages too were provided with fresh food plates every alternate day till 50% mortality was noticed in any of the cages, at which point all cages were provided fresh food plates supplemented with live yeast-acetic acid paste for three days following which eggs for starting next generation were collected. The eggs obtained from the two sister cages of a given population were mixed and redistributed into 160 vials.

Originally, the Control populations (also called as JB populations) were derived from IV populations^49^ and are described in detail in Prasad et al.^17^. The selected and control populations had been through 134 and 242 generations respectively at the time of being used in this study. To remove non-genetic parental effects which might appear due to the differences in maintenance regime, both the S and C populations were run through common rearing conditions for 1 generation at a moderate density of 50 eggs per 6 mL banana-jaggery media vial and 40 vials per population before being used in this study. The egg collection from the S and C populations were staggered by their developmental time difference to synchronize the emergence of adults (Fig. 1b). All adults emerging from each of the 40 vials of a given population were transferred to pre-labeled population cage with fresh SM plate. These populations are referred to as standardized flies^13,17^. Embryos for all the experiments were obtained from these standardized flies (SF).

### Generation of critical size adults and normal-sized adults

Synchronized eggs were collected from SF, evenly spread on agar–agar plates and incubated at SLC. Freshly hatched larvae (~ 22 h post-egg-laying) were harvested using a fine camel hair brush and transferred to Petri-plates (5.5 mm diameter, Tarson) with 2000 µL of Liquid Standard Media (LSM) at a density of 30 larvae per plate^13^. Twenty such plates per population were incubated at SLC. The larvae from all the 20 plates were re-harvested from LSM plates after 64 and 72 h (post-egg-lay) for S and C populations respectively, washed with Reverse osmosis (RO) water, rolled on tissue towel and randomly transferred (25 larvae per vial) to vials containing 6 mL non-nutritive agar–agar or SM and incubated at SLC^13^. At every 6 h interval, the emerging flies from these vials were collected, sorted according to their gender and held as virgins in pre-labeled holding vials with 6 mL SM till use in further assays. The adults that emerged from vials containing non-nutritive agar are referred to as critical-sized (CS) adults, while those that emerged from vials containing SM are referred to as normal-sized (NS) adults.

#### Macromolecule quantification during larval life

Glycogen and lipid content were estimated using Van Handel’s method^50^ and the protein content was estimated using Smith’s method^51^ with minor modifications.

All three macromolecules were quantified at 5 larval time points (Fig. 1b)—viz*.,* pre-critical stages (L2 to L3 transition stage—48 h for both C and S populations; Early L3 stage-56 h and 64 h for S and C populations respectively), critical size stage (64 h and 72 h for S and C) and post-critical stages (Late L3 stages—72 h and 104 h; before pupation L3—80 h and 112 h for S and C populations respectively) of larval life (Fig. 1b). All the sampling time intervals mentioned are from the time of transfer of newly hatched larvae to LSM plates. The detailed protocols used in the estimation of the macromolecules are as follows:

##### Glycogen estimation

Five randomly chosen larvae were homogenized in 400 µL of 2% Na_2_SO_4_. 80 µL of homogenate was aliquoted into 5 mL Eppendorf tube, to which 184 µL of Na_2_SO_4_ and 3736 µL of the (fresh) mixture of chloroform and methanol (1:1) was added. The tubes with the mix were centrifuged (Eppendorf, 5430R) at 14,000 r.p.m. for 10 min at 4 °C. The supernatant was discarded and the pellet was air-dried for 10 min. The pellet was resuspended in 2000 µL Anthrone reagent and heated at 90 °C in water-bath for 10 min. Aliquots were kept on ice for 5 min following which absorbance was measured at 625 nm on ELISA plate reader (ECIL micro scan, MS5605A). The assay was repeated in triplicate for both selected (n = 3) and control population (n = 3), and average of triplicate were used in statistical analysis.

##### Protein estimation

Precipitation assay was done before quantification of protein followed by BCA method of protein quantification^52^. Five randomly chosen larvae were homogenized in 400 µL of 2% Na_2_SO_4_. Then 80 µL of homogenate was aliquoted and 500 µL of 0.15% Deoxycholate was added to the aliquot. After an incubation period of 10 min on ice, 1000 µL 3 M Trichloroacetic acid (TCA) was added. The aliquots were centrifuged at 8500 r.p.m. (Eppendorf, 5430R) for 15 min at 4 °C. Protein was precipitated at the base of each aliquot. Pellets were washed with HCl and air-dried. BCA reagent was added to each of the pellets, resuspended and heated in water-bath at 60 °C for 10 min. Absorbance was recorded at 562 nm using ELISA plate reader (ECIL micro scan, MS5605A). The assay was repeated in triplicate for both selected (n = 3) and control population (n = 3), and average of triplicate were used in statistical analysis.

##### Lipid estimation

Five randomly chosen larvae were homogenized in 200 µL of PBS (1×). 1 mL of freshly prepared methanol and chloroform mixture (1:1) was added to the homogenate and vortexed. This was followed by centrifugation at 4 °C at 4000 r.p.m. (Eppendorf, 5430R). Two phases of the solution were separated. From the lower layer of solution, 200 µL of the solution was taken in an aliquot and was evaporated completely at 90 °C, using water-bath. 50 µL of H_2_SO_4_ was added post evaporation and incubated at 60 °C for 10 min, cooled on ice for 5–7 min., following which 1000 µL of Vanillin reagent was added and incubated at room temperature for 30 min. Absorbance was taken at 525 nm using ELISA plate reader (ECIL micro scan, MS5605A). The assay was repeated in triplicates per population and means of triplicate were used in statistical analysis.

All the biomolecules were converted to their energy equivalents^52^ and compared.

#### Adult life-history traits

##### Life-time fecundity and longevity

One day old virgins from holding vials were used in this assay. The flies were anaesthetized using CO_2_ and a female and male pair were transferred to fresh vials with 3 mL SM. A total of 20 pairs per treatment i.e. 20 pairs of critical sized flies and 20 pairs of normal sized flies were set up in both control and selected populations (Fig. 2a). Any fly that did not wake up within 1 h of transfer was replaced with a new fly of the same gender. Further, any fly that died within the first 24 h of set up was also replaced by a fresh fly. The pair of flies were transferred to fresh 3 mL SM vials every 24 h. The eggs laid in the preceding 24 h were counted under stereo zoom microscope (Carl Zeiss Binocular stereo zoom microscope, Stemi 305) and recorded. Census records were also maintained till the death of all flies. The average life-time fecundity and lifespan were estimated from this primary data (Fig. 2a).

##### Dry weight

Synchronized eggs from standardized flies were incubated at SLC. Five replicate vials per population with 50 eggs per vial were incubated in 6 mL of SM. Once blackening of pupae started, flies were observed at a time interval of six hours and adult flies were sorted on the basis of gender. They were pooled and transferred to pre-clean and dry vials as 10 flies per replicate population (n = 5), with a total of 50 flies per population per gender. Fresh weight of flies was taken and they were dried in oven at 70 °C for 36 h and weighed to obtain dry weight.

### Data analysis

In all cases except survival probability function, factorial-ANOVA, was performed using replicate population means and graphs were prepared using SPSS v. 22^53^. Since population means were used as units of analysis with selection, larval growth stage and fly gender as fixed variables and replication as a random variable^54^ only fixed-factor effects and interactions could be tested for significance^13,17^. The significance of adult survival probability curves was analyzed using Kaplan–Meier log-rank test^34^. Effect of female adult size on life-time fecundity was ascertained using linear regression analysis^54^.

### Ethics approval and consent to participate

Not applicable.

## Supplementary Information

Supplementary Information.

## Data Availability

Following is the link for reviewer: https://datadryad.org/stash/share/eG4xK0tSmnCIOTYYdBoRcEQrlFwIAE0HLrykA2dlGls.

## Abbreviations

SLC: Standard laboratory conditions
LSM: Liquid standard media
RO: Reverse osmosis
CS: Critical size
NS: Normal size
S: Selected
C: Control
LSP: Larval serum protein
CNS: Central nervous system
ANOVA: Analysis of variance
