# Supplementary material for: Fitness consequences of biochemical adaptation in Drosophila melanogaster populations under simultaneous selection for faster pre-adult development and extended lifespan
Source: Sci Rep. 2021 Aug 12;11:16434. doi: 10.1038/s41598-021-95951-2 (PMC8361192; doi:10.1038/s41598-021-95951-2)
Supplement: Supplementary file 1 — Supplementary Information. [file 41598_2021_95951_MOESM1_ESM.docx]

**Title**

Fitness consequences of biochemical adaptation in *Drosophila melanogaster* populations under simultaneous selection for faster preadult development and extended lifespan.

Khushboo Sharma and Mallikarjun N Shakarad*

Evolutionary biology laboratory, Department of Zoology, University of Delhi, Delhi-India

ORCID: 0000-0003-2278-3635 KS; 0000-0003-1134-3001 MS

Supplementary Figure S1- Energy equivalent (calories) per larva (a) glycogen content (b) protein content (c) lipid content.


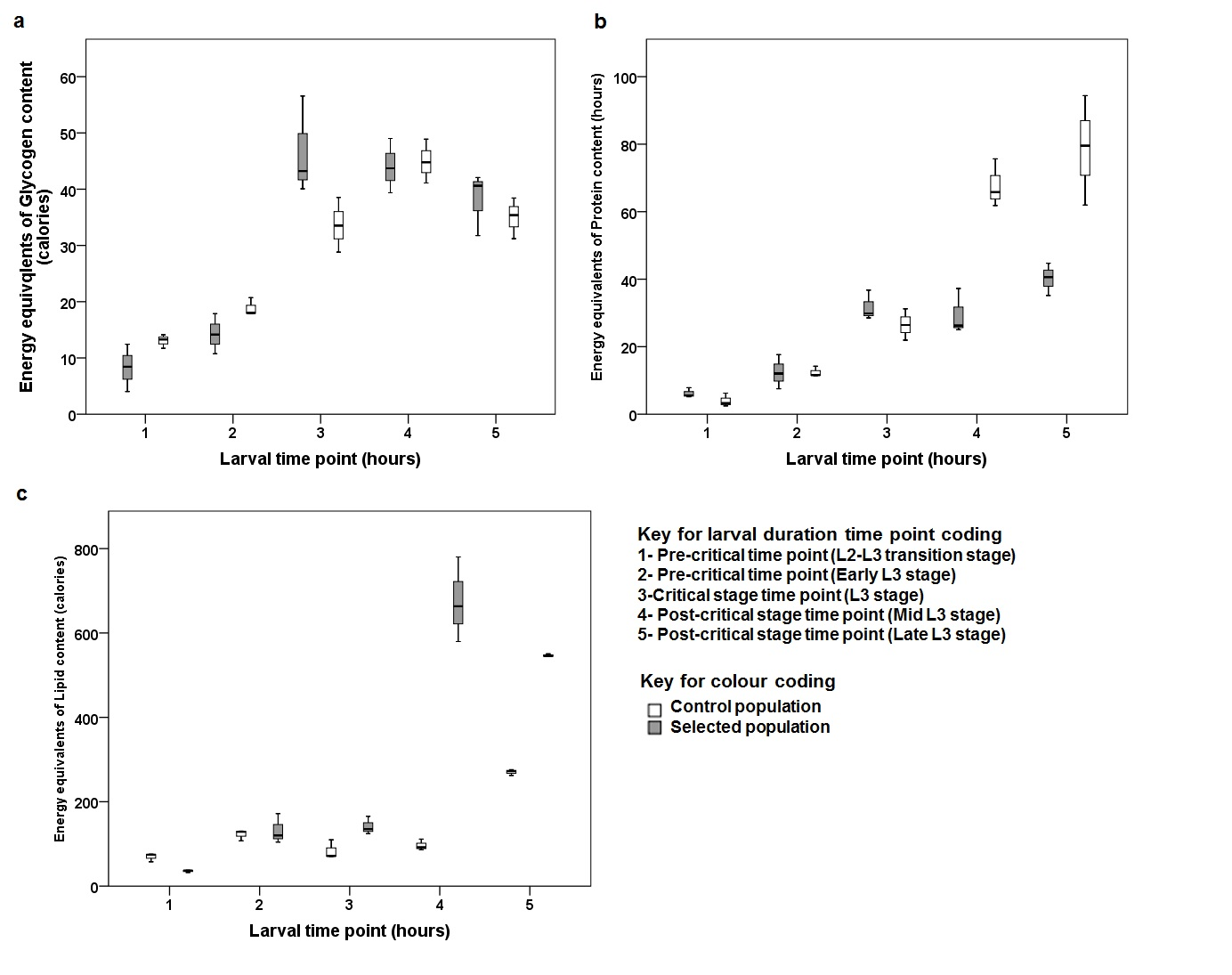


Supplementary Figure S2- Average value of Lifetime Longevity (a) females (b) males, in days described by boxplot graphs.


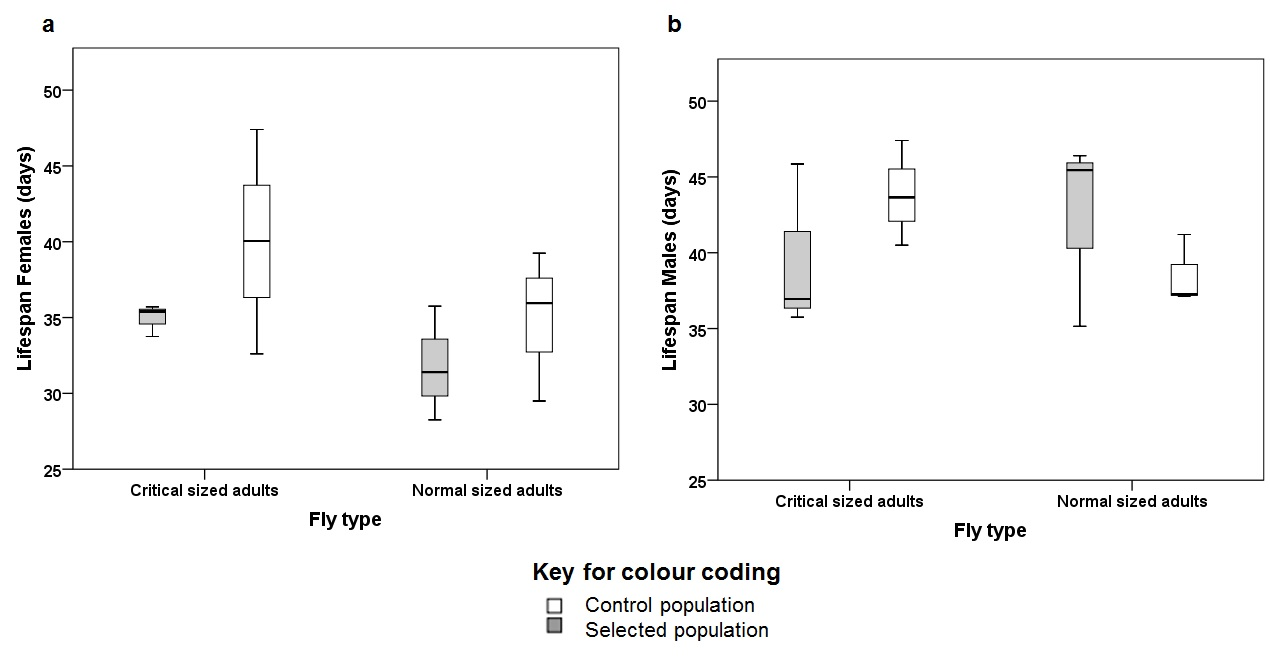


Supplementary Table S1- Bartlett test for Homogeneity of variance.

| **Assay/ Experiment** | **df** | **T- stat** | **p value** |
| --- | --- | --- | --- |
| **Biochemical Assay** |  |  |  |
| 1. Protein content | 9 | 16.21 | 0.0626 |
| 1. Glycogen content | 9 | 7.986 | 0.5355 |
| 1. Lipid content | 9 | 33.14 | 0.000125 |
| **Oviposition Assay** |  |  |  |
| Life time egg laying | 3 | 1.398 | 0.7058 |
| **Adult body size representative** |  |  |  |
| Dry weight of adults | **7** | 12.01 | 0.1002 |
|  |  |  |  |

Supplementary Table S2- Descriptive statistics of longevity in flies under selection for faster preadult development and extended longevity, and diet curtailing at critical size.

|  | | **FEMALES** | | | | | | | | | **MALES** | | | | | | | | | |
| --- | --- | --- | --- | --- | --- | --- | --- | --- | --- | --- | --- | --- | --- | --- | --- | --- | --- | --- | --- | --- |
|  | | **MEAN** | | | **MEDIAN** | | | **MAXIMUM** | | | **MEAN** | | | **MEDIAN** | | | **MAXIMUM** | | | |
| **Population & Fly type** | **N** | R1 | R2 | R3 | R1 | R2 | R3 | R1 | R2 | R3 | R1 | R2 | R3 | R1 | R2 | R3 | R1 | R2 | R3 |  |
| **S_CS_** | 60 | 33.75 | 35.7 | 35.4 | 33.5 | 33 | 37.5 | 61 | 74 | 62 | 36.95 | 45.85 | 35.75 | 41 | 43 | 33.5 | 70 | 79 | 69 |  |
| **S_NS_** | 60 | 28.25 | 35.75 | 31.4 | 25.5 | 31 | 32 | 67 | 73 | 52 | 45.45 | 46.4 | 35.15 | 46.5 | 43 | 36 | 59 | 74 | 59 |  |
| **C_CS_** | 60 | 47.4 | 40.05 | 32.6 | 54 | 41.5 | 34.5 | 64 | 68 | 59 | 47.4 | 43.65 | 40.5 | 48.5 | 42.5 | 41 | 68 | 69 | 70 |  |
| **C_NS_** | 60 | 39.25 | 35.95 | 29.5 | 44.5 | 36 | 27.5 | 59 | 58 | 54 | 41.2 | 37.25 | 37.15 | 46 | 37.5 | 35.5 | 60 | 63 | 62 |  |
| **F _population type_** | F_1,2_ | 0.889  NS  8.521  NS | | | 1.24  NS  18.75  <0.05 | | | 2.17  NS  3.005  NS | | | 0.008  NS  1.031  NS | | | 0.37  NS  0.48  NS | | | 0.617  NS  40.42  <0.05 | | | |
| **p _population type_** |  |  |  |  |  |  |  |  |  |  |  |  |  |  |  |  |  |  |  |  |
| **F _fly type_** | F_1,2_ |  |  |  |  |  |  |  |  |  |  |  |  |  |  |  |  |  |  |  |
| **p _fly type_** |  |  |  |  |  |  |  |  |  |  |  |  |  |  |  |  |  |  |  |  |

[*Each of the three replicate population (R1, R2 and R3) had 20 vials with one pair of male and female thus give a sample size of 60 flies/ population/ fly type/ gender.

NS- Non-significant difference; S_CS_ – Selected critical size population, S_NS_- Selected Normal sized population, C_CS_- Control critical sized population, C_NS_- Control Normal sized population].

Supplementary Table S3- Correlation between Dry weight (as body size representative) females with fecundity.

| **Dry weight of female flies** |  | **Dry weight of female flies** | **Fecundity** |
| --- | --- | --- | --- |
|  | Pearson correlation | 1 | 0.740** |
|  | Significance (2- tailed) |  | 0.006 |
|  | N | 12 | 12 |
| **Fecundity** |  |  |  |
|  | Pearson correlation | 0.740** | 1 |
|  | Significance (2- tailed) | 0.006 |  |
|  | N | 12 | 12 |

** Correlation is significant at the level of 0.01

Supplementary Table S4- Posthoc analysis- Tukey HSD values at level of significance < 0.05. Each column title is compared and analyzed with rest of the 4 time points, arranged in order of occurrence during larval life.

|  | **Larval time point** | | | | |
| --- | --- | --- | --- | --- | --- |
| **Energy Reserve content** | **Early L2** | **Late L2** | **Early L3** | **Mid L3** | **Late L3** |
| **Protein-content** | **.363** | **.363** | **.000** | **.000** | **.000** |
|  | **.000** | **.003** | **.003** | **.000** | **.000** |
|  | **.000** | **.000** | **.001** | **.001** | **.000** |
|  | **.000** | **.000** | **.000** | **.080** | **.080** |
| **Glycogen-content** | **.224** | **.224** | **.000** | **.000** | **.000** |
|  | **.000** | **.000** | **.000** | **.000** | **.000** |
|  | **.000** | **.000** | **.504** | **.504** | **.685** |
|  | **.000** | **.000** | **.685** | **.056** | **.056** |
| **Lipid-content** | **.013** | **.013** | **.059** | **.000** | **.000** |
|  | **.059** | **.955** | **.955** | **.000** | **.000** |
|  | **.000** | **.000** | **.000** | **.000** | **.000** |
|  | **.000** | **.000** | **.000** | **.799** | **.799** |
